# Association Between Riboflavin Intake and Suicidal Ideation: A Nationwide Study in Korea

**DOI:** 10.3390/nu17030449

**Published:** 2025-01-26

**Authors:** Hyejin Tae, Jeong-Ho Chae

**Affiliations:** 1 Stress Clinic, Health Promotion Center, Seoul St. Mary’s Hospital, College of Medicine, The Catholic University of Korea, Seoul 06591, Republic of Korea; 2 Department of Medicine, Graduate School, The Catholic University of Korea, Seoul 06591, Republic of Korea; 3 Department of Psychiatry, Seoul St. Mary’s Hospital, The Catholic University of Korea, Seoul 06591, Republic of Korea

**Keywords:** riboflavin, vitamin B, suicidal ideation, suicide, KNHANES

## Abstract

**Background/Objectives**: In recent years, there has been an increased interest in reducing suicide rates through dietary modification; however, the relationship between riboflavin intake and suicide risk remains unclear. This study aims to examine the association between dietary riboflavin and suicidal ideation. **Methods:** A total of 17,320 participants from the Korean National Health and Nutrition Examination Survey (KNHANES) 2014–2020 were included. Suicidal ideation was assessed using the ninth item of the Patient Health Questionnaire-9 (PHQ-9). Riboflavin intake was evaluated through dietary assessments. Multivariate logistic regression, restricted cubic spline (RCS) regression analysis, subgroup analysis, and interaction tests were conducted to explore the relationship between riboflavin intake and suicidal ideation. **Results**: There was a statistically significant association between riboflavin intake and suicidal ideation [OR (95%CI): 0.83 (0.77, 0.91), *p* < 0.001], after full adjustment for covariates. The linear trend test, using Q1 as the reference, showed ORs (95% CI) for Q2 and Q3 of 0.96 (0.81, 1.15) and 1.06 (0.80, 1.42), respectively. The RCS analysis revealed a non-linear pattern in the relationship between riboflavin intake and suicidal thoughts. This association was particularly significant among women and individuals younger than 60 years. Subgroup analyses and interaction tests indicated that the associations remained consistent across subgroups and were not influenced by factors other than anaerobic exercise. **Conclusions**: Our findings suggest a non-linear inverse relationship between riboflavin intake and suicidal ideation, with notable variations by sex and age. Modifying dietary riboflavin intake may be a crucial strategy for reducing suicide risk.

## 1. Introduction

Suicide has long been one of the leading causes of death worldwide. According to WHO estimates, more than 700,000 people are lost due to suicide every year, translating to one person taking their own life approximately every 40 s [1]. In particular, South Korea reached 27.3 suicide deaths per 100,000 in 2023, which is more than double the OECD average of 11.0 deaths per 100,000 and ranking as the number one suicide rate among the members of the OECD [2]. Suicide is the fifth most common cause of death in South Korea, and it is the leading cause of death for those aged 10 to 39 years [3]. Furthermore, suicide creates profound psychosocial and economic burdens on individuals, families, and communities [4]. This result suggests that suicide is a major public health problem, and it has become important to identify various risk factors for suicide.

Although many factors are related to suicide [5], the role of a healthy lifestyle in the prevention of suicide has received increasing interest. When it comes to diet, a modifiable factor that could affect mental health [6], a few studies have explored specific nutrients and foods in relation to suicide risk. A cross-sectional study reported that fruits, vegetables, and meat were significantly under-consumed by the individuals with a lifetime history of attempted suicide [7]. In a population-based prospective study, a dietary pattern characterized by high consumption of vegetables, fruits, soy products, potatoes, seaweed, mushrooms, and fish was significantly related to a reduction in suicide risk [8]. Other foods and nutrients such as tryptophan (included in meat) [9] and coffee [10] have been linked to a decreased risk of suicide.

In terms of micronutrients in food, riboflavin, known as vitamin B2 has emerged as a “neurotropic” vitamin since it has a remarkable influence on the normal function of both central and peripheral nervous systems [11]. Riboflavin forms coenzymes like flavin mononucleotide (FMN) and flavin adenine dinucleotide (FAD) are essential rate-limiting components in most cellular enzymatic reactions [12]. Since the metabolism of fatty acids in brain lipids is facilitated by flavoproteins [13], a deficiency in riboflavin would be harmful to brain function. Several neurological and psychiatric diseases, such as depression, migraines, and peripheral neuropathy, have been associated with riboflavin deficiencies [14]. Although there is some research on the relationship between riboflavin consumption and the risk of depression or mental disorders [15,16], no research to date has explored the association between riboflavin consumption and suicidal risk in the general population. Therefore, we investigated whether dietary intake of riboflavin is independently related to suicidal ideation, independent of depression, utilizing a large-scale population-based cohort of Korean adults.

## 2. Materials and Methods

### 2.1. Data Source and Study Population

**Figure 1:**
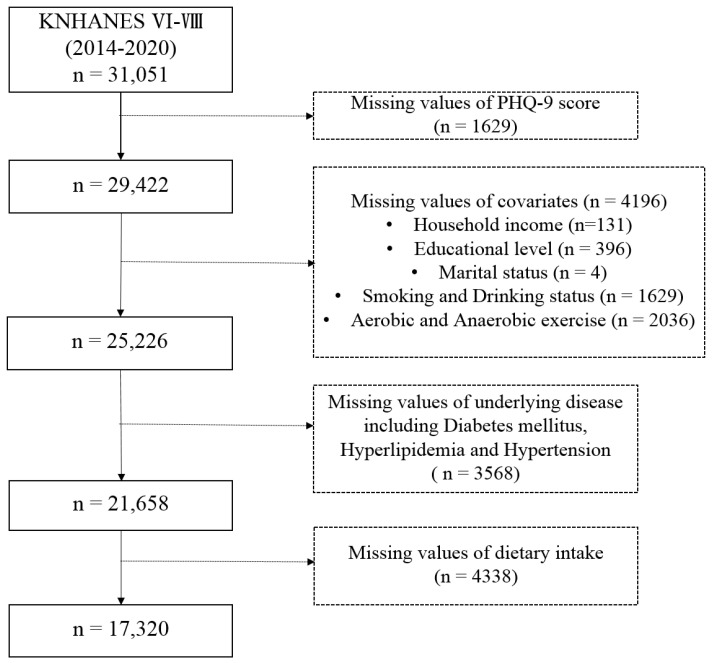
Flowchart of sample selection from KNHANES 2014–2020.

We employed a nationwide population-based database from the KNHANES (Korean National Health and Nutrition Examination Survey), periodically conducted by the Korea Centers for Disease Control and Prevention to investigate the health and nutritional status of Korea utilizing a nationally representative population of the Koreans. The KNHANES is composed of three component surveys: a health interview, a nutrition survey, and physical examinations [17]. The surveys involve the collection of information from individuals including demographic characteristics, diet and health-related variables, anthropometric measures, and biochemical profiles. The sample for this study encompasses data from the sixth to eighth cycles of the KNHANES study (2014–2020). From 31,051 eligible individuals, we excluded 13,731 persons for the following reasons: missing information regarding PHQ-9 (*n* = 1629), missing data on covariates (*n* = 4196), missing information about an underlying disease (*n* = 3568), and incomplete dietary data (*n* = 4338), leaving 17,320 individuals for analysis (Figure 1). All KNHANES surveys obtained approval from the Institutional Review Board (IRB) of the Korea Centers for Disease Control and Prevention. All participants provided signed informed consent forms before their enrollment (IRB: 2013-12EXP-03-5C, 2018-01-03-P-A, 2018-01-03-2C-A).

### 2.2. Assessment of Riboflavin Intake

Riboflavin and other dietary intakes of participants were collected through a 24-h dietary recall interview, which was conducted on a single day in each survey period to assess dietary variables. Trained researchers administered face-to-face interviews in the participants’ homes, wherein participants reported all the food and drinks consumed in the previous 24 h. Daily energy intake, nutrients, and other food components were analyzed using the National Standard Food Composition Table published by the Rural Development Administration [18].

### 2.3. Assessment of Psychological Symptoms

The Patient Health Questionnaire-9 (PHQ-9) was performed to assess the depressive symptoms of participants, which was used for depression screening in general and clinical populations [19]. It consisted of nine items rated on a 4-point Likert scale (0–3). The total score ranged from 0 to 27, and a total score of 11 or higher represented clinically significant depressive symptoms, which was applied in the present study [20]. The Korean version of the PHQ-9 showed good validity and reliability [21].

Suicidal ideation was assessed using item 9 from the PHQ-9, which asks, “How often have you had thoughts that you would be better off dead, or of hurting yourself in some way within the last two weeks?” Response options are “never”, “several days”, “more than one week”, and “everyday”. Respondents who answered “several days,” “more than one week”, and “everyday” were categorized as individuals who had suicidal ideation.

### 2.4. Assessment of Covariates

Various sociodemographic characteristics (age, sex, marital status, educational level, household income) and behavioral factors (smoking status, drinking status, aerobic exercise, and anaerobic exercise) were considered in the present analysis. Participants were classified into four education-level groups as follows: less than elementary school, middle school, high school, and college or more. Monthly household income was divided into four groups by quartiles as follows: low, medium-low, medium-high, and high. Existing smokers were defined as those who had smoked at least 100 cigarettes over their lifetime and were currently smoking. Drinkers referred to those who had consumed alcohol at least once a month in the last year. Aerobic exercise was defined by responses to the question, “How many days and time do you spend in physical activity such as walking?” “How many days and time do you spend in strength training, such as push-ups, sit-ups, or lifting barbells or dumbbells, to develop muscles?” was an example of a question about anaerobic exercise. Participants were classified as ’active’ if they reported engaging in physical activity at least 5 times a week and more than 30 min each time. In contrast, participants were classified as ‘inactive’ if they failed to meet the physical activity guidelines.

BMI was calculated as the individual’s weight in kilograms divided by the square of their height in meters. Participants with a BMI of ≥25 kg/m^2^ were considered obese by the recommended BMI cut-off for Asian populations. Hypertension was defined as systolic blood pressure values ≥ 140 mmHg and/or diastolic blood pressure values ≥ 90 mmHg, use of antihypertensive agents, or reported diagnosis of hypertension. Diabetes was defined as fasting blood glucose concentration ≥ 126 mg/dL, use of anti-diabetic drugs, or reported diagnosis of diabetes. Hyperlipidemia was defined as the presence of one or more of the following conditions: total cholesterol ≥ 240 mg/dL, triglyceride ≥ 150 mg/dL, high-density lipoprotein cholesterol ≤ 40 mg/dL, low-density lipoprotein cholesterol ≥ 130 mg/dL, or use of lipid-lowering drugs. All blood samplings were performed on participants who had fasted for at least 8 h. Fasting glucose (mg/dL) levels were measured using the Labospect 008AS autoanalyzer (Hitachi, Tokyo, Japan). Cholesterol and TG (mg/dL) levels were analyzed by enzymatic methods using Labospect 008AS (Hitach, Japan).

### 2.5. Statistical Analyses

Since KNHANES employed sample weighting, stratification, and cluster variables, all statistical analyses in this study were performed utilizing the complex sampling design of the KNHANES database to enhance the representativeness of the sample and the accuracy of the estimates. The baseline characteristics of the study population are described according to the tertiles of riboflavin intake. The demographic and clinical features of the participants were shown as means (standard deviation [SD]) for continuous variables and as numbers (%) for categorical variables. Differences in characteristics across tertiles were analyzed using a one-way analysis of variance (ANOVA) (continuous variables) and chi-square test (categorical variables). Given that suicidal ideation was a dichotomous variable, we performed multivariable logistic regression models to examine the association between covariates and suicidal ideation. The crude model (Model 1) was unadjusted. Model 2 accounted for age and sex. Model 3 was adjusted for age, sex, marital status, educational level, household income level, smoking status, drinking status, aerobic exercise, anaerobic exercise, hypertension, dyslipidemia, diabetes mellitus, depression, BMI, thiamine intake, niacin intake, and daily energy intake. A linear trend test was conducted to confirm the stability of the results. We applied restricted cubic spline (RCS) with knots positioned at the 5th, 35th, 65th, and 95th percentiles of the riboflavin distribution to model the dose-response relationship between riboflavin intake and suicidal ideation, allowing for a more flexible approach for potential nonlinear patterns. The RCS models were further stratified by sex, age, and BMI. We performed subgroup analyses to explore the link between riboflavin consumption and suicidal ideation across different subgroups. Interaction tests were used to evaluate the effect of riboflavin intake on suicidal ideation relevant to several stratified covariates. The data analyses were performed utilizing R software (version 4.2.3), and a two-sided *p* value of <0.05 was stated as statistically significant.

## 3. Results

### 3.1. Baseline Characteristics of the Study Population

The baseline characteristics of the study participants according to the tertiles of riboflavin intake in Table 1. Among the study population, the proportion of men and women was 41.83% and 58.17%, respectively. A significant proportion of participants identified as being married or living with a partner (83.46%), and 36.78% had attained a college educational level. In contrast to the lowest tertile of riboflavin, subjects in the highest tertile group were found to have a higher likelihood of being younger, male, and single or separated. They also exhibited higher education levels, higher household income, and higher daily energy intake than the other groups. The prevalence of suicidal ideation, depressive symptoms, and comorbidities including hypertension, diabetes, and hyperlipidemia showed a significant decrease as riboflavin intake increased.

### 3.2. Association Between Riboflavin Intake and Suicidal Ideation

The association of each covariate with suicidal ideation is presented in Table 2. Women were at increased risk of developing suicidal ideation compared to men (OR: 1.791, 95% CI: 1.598, 2.008). Confounding factors such as marital status, smoking status, hypertension, diabetes, hyperlipidemia, depression, and BMI (≥25 kg/m^2^) were positively associated with suicidal ideation. On the other hand, variables such as educational level, household income, aerobic and anaerobic exercise, omega-3 fatty acid intake and thiamine intake were negatively related to suicidal ideation.

**Figure 2:**
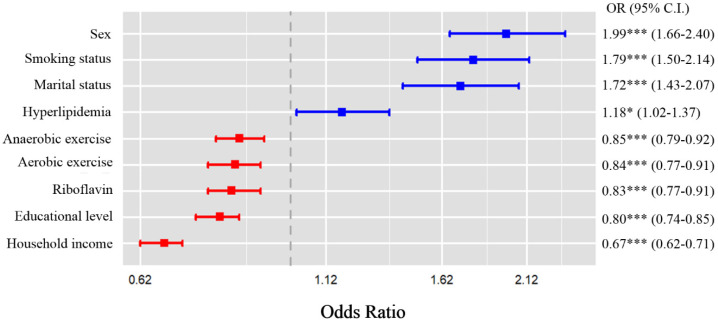
Results from the multivariate logistic analysis for suicidal ideation. * *p* < 0.05, *** *p* < 0.001. Blue line: OR > 1.00, red line: OR < 1.00.

Multivariable logistic regression analysis was performed to explore the relationship between riboflavin intake and suicidal ideation (Table 3). After fully adjusting for confounders in Model 3, the association between riboflavin intake and suicidal ideation showed a significant negative association (Model 3; OR: 0.83, 95% CI: 0.77, 0.91). The summarized results of significant covariates for multivariate logistic analyses were presented in Figure 2. Women had 99% higher odds of having suicidal ideation as compared to men (OR: 1.99, 95% CI: 1.66, 2.40). Smokers and married individuals had 1.79 times and 1.72 times higher OR for suicidal ideation than non-smokers and single individuals, respectively. In contrast, high household income and high educational level predicted statistically significant decreases in suicidal ideation. Each one-unit increase in riboflavin intake was associated with a 17% decrease in the likelihood of suicidal ideation, which was lower odds compared to aerobic exercise (OR: 0.84, 95% CI: 0.77, 0.91) or anaerobic exercise (OR: 0.85, 95% CI: 0.79, 0.92).

Multivariable logistic regression stratified by sex revealed a significant association between riboflavin intake and suicidal ideation in women (OR: 0.78, 95% CI: 0.70, 0.87, Table 3) but not in men (OR: 0.95, 95% CI: 0.82, 1.11). The age-based subgroup analyses revealed that the relationships were statistically significant in those aged <60 years (OR: 0.82, 95% CI: 0.69, 0.97) and those aged ≥60 years (OR: 0.88, 95% CI: 0.79, 0.97), respectively. Additionally, the relationships were similarly significant in those with a BMI < 25 kg/m^2^ (OR: 0.80, 95% CI: 0.71, 0.90) and those with a BMI ≥ 25 kg/m^2^ (OR: 0.88, 95% CI: 0.78, 0.99).

To further explore the association between riboflavin intake and suicidal ideation, we conducted a linear trend test based on tertiles of riboflavin consumption. Using Q1 as the reference, the ORs (95% CI) for Q2 and Q3 were 0.96 (0.81, 1.15) and 1.06 (0.80, 1.42), respectively, with the *p* value of the linear trend test being 0.230, suggesting a potential non-linear relationship between riboflavin intake and suicidal ideation. That meant the relationship was not simply linear, but with bends and curves, so another statistical method used to model a non-linear relationship was needed.

### 3.3. Analysis of Restricted Cubic Spline (RCS) Regression

**Figure 3:**
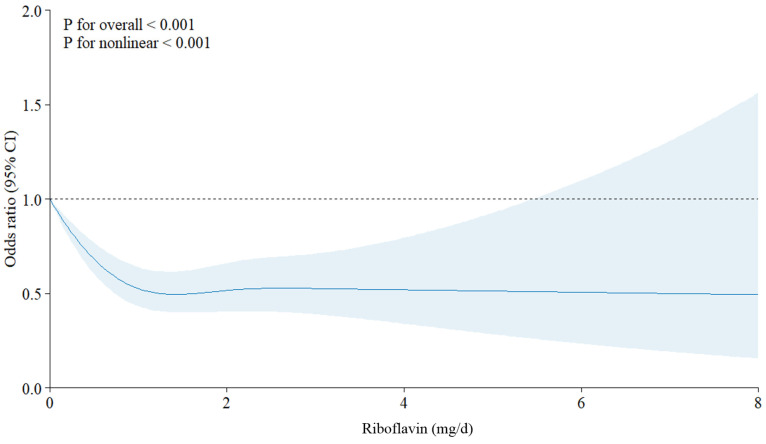
Association between riboflavin and suicidal ideation based on a restricted cubic spline model. Shaded areas represent 95% confidence intervals. The analysis was adjusted for several factors including age, sex, BMI, marital status, educational level, household income level, smoking status, drinking status, aerobic exercise, anaerobic exercise, hypertension, dyslipidemia, diabetes mellitus, depression, body mass index, thiamine intake, niacin intake, and daily energy intake.

**Figure 4:**
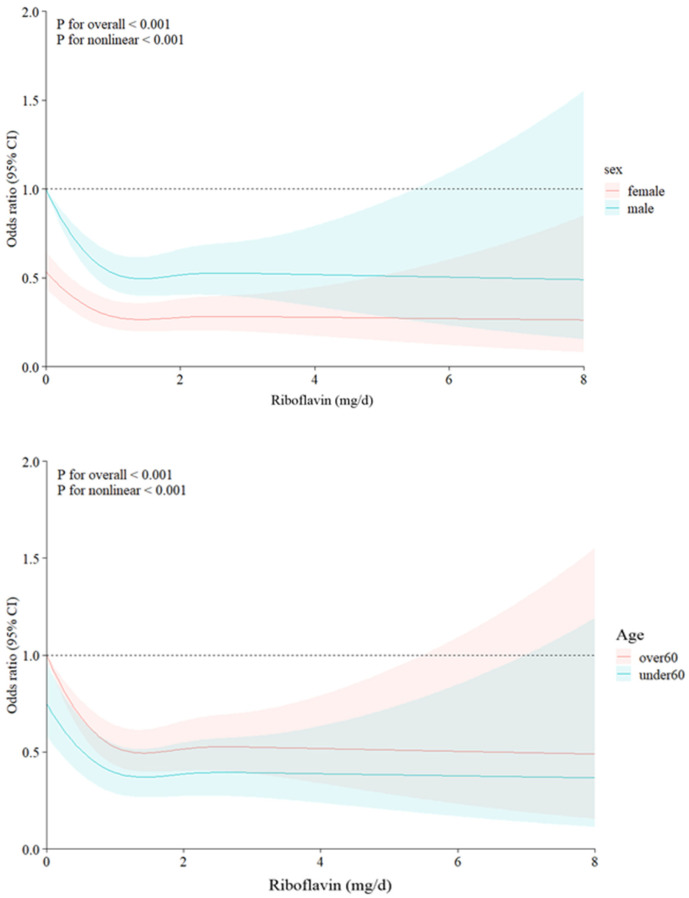

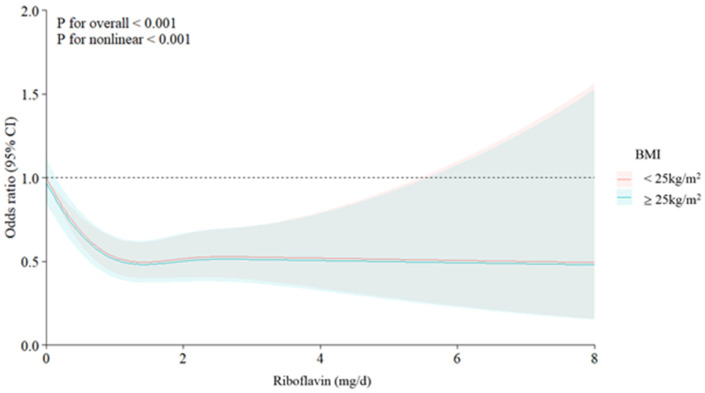
Association between riboflavin and suicidal ideation using a restricted cubic spline model, stratified by sex, age, and BMI (Body mass index). Shaded areas indicate 95% confidence intervals. This association was adjusted for age, sex, BMI, marital status, educational level, household income level, smoking status, drinking status, aerobic exercise, anaerobic exercise, hypertension, dyslipidemia, diabetes mellitus, depression, thiamine intake, niacin intake, and daily energy intake.

We applied restricted cubic spline (RCS) to model the dose-response relationship between riboflavin intake and suicidal ideation, allowing for a more flexible exploration of potential non-linear patterns. Results of the RCS analysis indicated a non-linear association (*p* for non-linearity < 0.001) between riboflavin intake and suicidal ideation, and this association was approximately L-shaped (Figure 3). We performed subgroup RCS analyses to explore the association between riboflavin intake and suicidal ideation that existed in subgroups defined by sex, age, and BMI (Figure 4). The results showed similar characteristics to the overall trend (*p* for non-linearity < 0.001), with stronger associations among females and those aged less than 60 years.

### 3.4. Association Between Riboflavin Intake and Suicidal Ideation in Different Subgroups

Subgroup analyses and interaction tests were based on variables including age, sex, BMI, aerobic exercise status, anaerobic exercise status, hypertension status, diabetes status, and hyperlipidemia status. As presented in Table 4, interaction tests showed that the association between riboflavin intake levels and suicidal ideation remained stable when stratified by other covariates (*p* for interaction < 0.05) except for the variable of anaerobic exercise status (*p* for interaction = 0.0284).

## 4. Discussion

To the best of our knowledge, this is the first study to investigate the association between dietary riboflavin intake and suicidal ideation in the general population. The results showed that levels of riboflavin intake were inversely related to the presence of suicidal ideation, after adjusting for covariates such as depressive symptoms. Our analysis unveiled an L-shaped nonlinear relationship between riboflavin consumption and suicidal thoughts. In addition, stratified analyses by sex, age, and BMI showed population differences. Subgroup analyses and interaction tests revealed that the relationships remained consistent across different subgroups and were not affected by other variables except for anaerobic exercise. The negative association between riboflavin intake and suicidal ideation was more pronounced in those who engage regularly in anaerobic exercise. Our study may be able to suggest a reference for the decreasing of suicidal ideation through dietary modifications.

The inverse relationship reported in the present study between serum riboflavin levels and suicide risk is in line with previous findings of the inverse relationship between riboflavin consumption and depression, which is a main precondition of suicide [22]. An inverted J-shaped association between riboflavin consumption and postpartum depression was found in a prospective cohort study [23]. An observational study reported higher intake of riboflavin was related to a lower severity of depression [24]. Similarly, a meta-analysis of six epidemiologic studies showed a significant inverse association between riboflavin intake and depressive symptoms [25]. Although low riboflavin consumption may be linked to a high prevalence of suicide as a consequence of depression, our study found a significant decreased risk of suicidal ideation with the increased consumption of riboflavin, even after adjusting for depressive symptoms.

Even though the mechanisms underlying the relationship between riboflavin and suicidal ideation remain unclear, several lines of evidence indicated the role of riboflavin in the pathogenesis of suicidal ideation. The first is hyperhomocysteinemia. Several studies reported that elevated homocysteine levels may be linked to increased suicide severity [26,27]. Homocysteine is essential for the biosynthesis of monoamine neurotransmitters such as serotonin, dopamine, and norepinephrine. Changes in homocysteine metabolism can affect the formation of these neurotransmitters, which have a profound influence on mood regulation and suicidality [28]. The benefits of riboflavin may be explained by the activation of riboflavin coenzymes in the remethylation and transsulfuration of homocysteine [29], and previous studies showed a negative relationship between riboflavin and homocysteine levels [30]. The second is oxidative stress. The significant association between suicidal ideations and oxidative stress component levels (oxidative stress index, nicotinamide adenine dinucleotide phosphate (NADPH) oxidase, and advanced oxidation protein products) was identified [31]. Riboflavin is essential for the synthesis, conversion, and recycling of other vitamins and proteins related to oxygen transport. Its derivatives show antioxidant activities and are cofactors in the metabolism of fatty acids in brain lipids [32]. As an antioxidant, riboflavin may help to reduce the damage to the nervous system caused by oxidative stress and influence suicidal ideation through the reductive oxidative reaction of riboflavin itself, its action on glutathione’s redox cycle, and antioxidant enzyme activity [33]. In addition, evidence shows that anaerobic exercise has great potential to enhance antioxidant defenses [34] and increase BDNF levels, which regulates the expression of antioxidant molecules [35]. In this respect, anaerobic exercise might affect the relationship between riboflavin intake and suicidal ideation through its antioxidant capacity. The third is neuroinflammation. To date, several studies indicated inflammation, mediated by both pro-inflammatory cytokines (IL-1, IL-6, and TNFα) and abnormally activated immune cells (monophagocytes and glial cells) might contribute to the pathophysiology of suicide [36,37]. These inflammatory factors tend to be overactive in suicidal subjects and related to the severity of suicidal ideation [38]. Riboflavin has an anti-inflammatory effect by decreasing NF-κB synthesis, leading to reduced nitric oxide and TNFα levels, and regulates the increased levels of the anti-inflammatory cytokine [39].

The current study has some notable strengths that contribute to the significance of the findings. First, this study is the first to demonstrate the relationship between riboflavin consumption levels and suicidal ideation, providing novel insights into the probability of dietary modification for suicide prevention. Second, we focused on riboflavin derived from food consumption, providing a practical indicator. Third, the use of KNHANES data, which employs a meticulous multistage stratified cluster sampling design, provides a basis for the generalizability and applicability of the results to the broader population. Fourth, this study has considered potential confounders including socio-demographic characteristics, dietary intake, and various comorbidities, especially depressive symptoms, allowing for a more exact evaluation of the link between riboflavin levels and suicidal thoughts. Consequently, our findings could provide useful evidence for public health strategies aimed at preventing suicide.

This study had several limitations that may serve as suggestions for future research. First, we cannot investigate a causal association between riboflavin consumption and suicidal thoughts due to the use of a cross-sectional design. Conducting randomized controlled trials or prospective investigations would provide more robust evidence for establishing the preventive effect of riboflavin. Second, the assessment of suicidal ideation and depressive symptoms was conducted using the PHQ-9, which is a self-reporting instrument. Self-reported data can often be inaccurate due to recall bias or social desirability bias. Additionally, PHQ-9 is useful for screening, but not for the diagnosis of current major depressive episodes and the assessment of the long-term risk of depression [40]. However, this method is widely accepted, and its high sensitivity and specificity have been clinically validated [41]. Third, although the PHQ-9’s item 9 has been used in prior research to measure suicidal ideation [42], its extensive definition may influence how the study evaluates the items’ link to suicidal ideation. Fourth, dietary intake was evaluated at a single time point, and the measurements may not reflect usual dietary patterns accurately. Assessing dietary intake repeatedly over a long period of time would result in better estimates of exposure. Fifth, we could not estimate the effect of other psychiatric disorders on suicidal ideation because the KNHANES did not contain sufficient information. For example, several studies showed that personality disorders may influence the type of diet and consequently the prognosis of psychiatric conditions including suicidality [43]. Sixth, the use of vitamin supplements was not considered in our study. A cross-sectional study suggested that low consumption of antioxidant food has been reported among suicide attempters compared with non-attempters, and these differences were reduced by adjustment for vitamin supplementation [44]. The insufficient data on vitamin supplements could overestimate the riboflavin intake effect on suicidality. When taking into account that the consumption of vitamin supplements has increased, evaluating the effect of vitamin supplements associated with suicidal thoughts deserves further study.

Based on the analysis conducted, we revealed a nonlinear inverse association between dietary riboflavin intake and suicidal ideation and further explored differences between sex, age, and BMI. Although more research is needed to identify the causality of this relationship, dietary modification to intervene in the consumption of riboflavin may be an important strategy for decreasing suicide risk. Further research is required to determine the precise dosage of riboflavin through dietary intake needed to achieve anticipated therapeutic effects for suicide prevention.

## 5. Conclusions

In conclusion, our study discovered that riboflavin intake levels were inversely associated with the presence of suicidal ideation, even after adjusting for covariates including depressive symptoms. Our analysis revealed an L-shaped pattern in the relationship between riboflavin consumption and suicidal thoughts. Moreover, the impact of riboflavin consumption on reducing the risk of suicidal ideation was more significant in females, individuals under 60 years, and those with a BMI < 25 kg/m^2^. Subgroup analyses and interaction tests confirmed that these relationships remained consistent across various subgroups and were not influenced by other variables, except for anaerobic exercise. The inverse association between riboflavin intake and suicidal ideation was more pronounced among individuals who engage regularly in anaerobic exercise. Our findings underscore the importance of dietary interventions in riboflavin consumption as a potential strategy to decrease suicide risk. If consolidated by additional evidence, our results could suggest the benefits of riboflavin modification to help clinicians in decision-making and policymakers in improving public health.

## Figures and Tables

**Table 1:** Characteristics of study participants and riboflavin intake tertile.

Variable	Q1 (*n* = 5774)	Q2 (*n* = 5773)	Q3 (*n* = 5773)	*p*-Value
Age (years)	54.6 ± 17.3	51.6 ± 16.4	47.1 ± 15.1	<0.001
Sex (%)				<0.001
Male	1891 (32.8)	2247 (38.9)	3107 (53.8)	
Female	3883 (67.2)	3526 (61.1)	2666 (46.2)	
Marital status (%)				<0.001
Married/Living with partner	4887 (84.6)	4919 (85.2)	4649 (80.5)	
Widowed/Never married/ Divorced/Separated	887 (15.4)	854 (14.8)	1124 (19.5)	
Educational Level (%)				<0.001
Elementary or Less	1625 (28.1)	1247 (21.6)	539 (9.3)	
Middle School	672 (11.6)	666 (11.5)	436 (7.6)	
High School	1747 (30.3)	1866 (32.3)	2151 (37.3)	
College or More	1730 (30.0)	1994 (34.5)	2647 (45.9)	
Household Income (%)				<0.001
Low	1436 (24.9)	1049 (18.2)	598 (10.4)	
Medium-Low	1440 (24.9)	1472 (25.5)	1310 (22.7)	
Medium-High	1460 (25.3)	1646 (28.5)	1798 (31.1)	
High	1438 (24.9)	1606 (27.8)	2067 (35.8)	
Smoking Status				< 0.001
No	4009 (69.4)	3869 (67.0)	3288 (57.0)	
Yes	1765 (30.6)	1904 (33.0)	2485 (43.0)	
Drinking Status (%)				<0.001
No	3082 (53.4)	2849 (49.4)	2283 (39.5)	
Yes	2692 (46.6)	2924 (50.6)	3490 (60.5)	
Aerobic Exercise (%)				<0.001
<1 day/week	1205 (20.9)	1056 (18.3)	901 (15.6)	
1–3 days/week	1629 (28.2)	1677 (29.0)	1684 (29.2)	
4–7 days/week	2940 (50.9)	3040 (52.7)	3188 (55.2)	
Anaerobic exercise (%)				0.004
<1 day/week	2522 (43.7)	4367 (75.6)	2780 (48.2)	
1–3 days/week	664 (11.5)	746 (12.9)	949 (16.4)	
4–7 days/week	2588 (44.8)	660 (11.4)	2044 (35.4)	
Hypertension (%)				<0.001
Normal	2292 (39.7)	2582 (44.7)	2816 (48.8)	
Prehypertension	1326 (23.0)	1404 (24.3)	1477 (25.6)	
Hypertension	2156 (37.3)	1787 (31.0)	1480 (25.6)	
Diabetes (%)				<0.001
Normal	3106 (53.8)	3493 (60.5)	3733 (64.7)	
Prediabetes	1723 (29.8)	1509 (26.1)	1478 (25.6)	
Diabetes	945 (16.4)	771 (13.4)	562 (9.7)	
Hyperlipidemia (%)				<0.001
No	4273 (74.0)	4603 (79.7)	4677 (81.0)	
Yes	1501 (26.0)	1170 (20.3)	1096 (19.0)	
Depression (%)				0.001
No	5399 (93.5)	5442 (94.3)	5536 (95.9)	
Yes	375 (6.5)	331 (5.7)	237 (4.1)	
Suicidal Ideation (%)				0.003
No	5367 (93.0)	5402 (93.6)	5527 (95.7)	
Yes	407 (7.0)	371 (6.4)	244 (4.3)	
Body Mass Index, kg/m^2^	24.0 ± 3.6	23.8 ± 3.5	24.1 ± 3.5	0.13
Daily Energy Intake, kcal/d	1438.2 ± 559.2	1808.7 ± 613.1	2586.7 ± 958.7	<0.001
Carbohydrates, g/d	234.7 ± 94.4	286.4 ± 104.3	360.6 ± 133.6	<0.001
Total Dietary Fiber, g/d	19.7 ± 10.7	24.7 ± 12.4	32.5 ± 16.1	<0.001
Protein, g/d	48.1 ± 22.1	62.0 ± 25.4	98.2 ± 44.4	<0.001
Fat, g/d	27.4 ± 18.8	37.8 ± 24.2	66.3 ± 41.8	<0.001
Polyunsaturated Fatty Acid, g/d	7.7 ± 5.9	10.1 ± 7.3	16.5 ± 11.2	<0.001
Omega-3 Fatty Acid, g/d	1.3 ± 1.4	1.6 ± 1.6	2.5 ± 2.3	<0.001
Omega-6 Fatty Acid, g/d	6.4 ± 5.1	8.5 ± 6.3	13.9 ± 9.8	<0.001
Retinol, μg/d	66.2 ± 94.5	95.1 ± 156.9	249.0 ± 659.6	<0.001
Beta-Carotene, μg/d	2049.1 ± 2114.9	2972.6 ± 3277.2	4275.2 ± 5405.1	<0.001
Thiamine, mg/d	0.9 ± 0.5	1.4 ± 0.7	2.0 ± 1.1	<0.001
Riboflavin (mg/d)	0.8 ± 0.4	1.2 ± 0.4	2.5 ± 0.8	<0.001
Niacin (mg/d)	9.0 ± 4.6	12.7 ± 5.7	19.1 ± 9.7	<0.001
Ascorbic Acid (mg/d)	44.8 ± 47.7	75.1 ± 79.4	101.0 ± 126.7	<0.001

Values are presented as mean ± standard deviation or number (%). Statistical significance was determined using a one-way analysis of variance (ANOVA) or Chi-square tests.

**Table 2:** Association of covariates with suicidal ideation.

	OR (95% CI)	*p*
Age	1.031 (1.028, 1.034)	<0.001
Sex (%)		
Male	1.00	
Female	1.791 (1.598, 2.008)	<0.001
Marital Status (%)		
Married/Living with Partner	1.00	
Widowed/Never Married Divorced/Separated	2.527 (2.195, 2.910)	<0.001
Educational Level (%)		
Elementary or Less	1.00	
Middle School	0.871 (0.728, 1.041)	0.129
High School	0.863 (0.757, 0.984)	0.028
College or more	0.535 (0.461, 0.620)	<0.001
Household income (%)		
Low	1.00	
Medium-Low	0.352 (0.306, 0.404)	<0.001
Medium-High	0.253 (0.219, 0.292)	<0.001
High	0.162 (0.137, 0.192)	<0.001
Smoking status		
No	1.00	
Yes	1.591 (1.424, 1.777)	<0.001
Drinking status (%)		
No	1.00	
Yes	1.078 (0.967, 1.201)	0.177
Aerobic exercise (%)		
<1 day/week	1.00	
1–3 days/week	0.469 (0.406, 0.543)	<0.001
4–7 days/week	0.503 (0.442, 0.573)	<0.001
Anaerobic exercise (%)		
<1 day/week	1.00	
1–3 days/week	0.403 (0.338, 0.481)	<0.001
4–7 days/week	0.409 (0.360, 0.466)	<0.001
Hypertension (%)		
Normal	1.00	
Prehypertension	1.196 (1.036, 1.381)	0.015
Hypertension	1.643 (1.451, 1.860)	<0.001
Diabetes (%)		
Normal	1.00	
Prediabetes	1.068 (0.934, 1.220)	0.338
Diabetes	1.573 (1.348, 1.836)	<0.001
Hyperlipidemia (%)		
No	1.00	
Yes	1.325 (1.164, 1.508)	<0.001
Depression (%)		
No	1.00	
Yes	45.001 (39.407, 51.389)	<0.001
Body mass index, kg/m^2^	1.062 (1.049, 1.075)	<0.001
Daily energy intake, kcal/d	1.000 (1.000, 1.000)	<0.001
Carbohydrates, g/d	0.999 (0.998, 0.999)	<0.001
Total Dietary Fiber, g/d	0.992 (0.988, 0.997)	0.001
Proteins, g/d	0.988 (0.986, 0.990)	<0.001
Fats, g/d	0.986 (0.983, 0.988)	<0.001
Polyunsaturated Fatty Acids, g/d	0.954 (0.945, 0.963)	<0.001
Omega-3 Fatty Acids, g/d	0.862 (0.825, 0.901)	<0.001
Omega-6 Fatty Acids, g/d	0.947 (0.937, 0.957)	<0.001
Retinol, μg/d	0.998 (0.997, 0.998)	<0.001
Beta-carotene, μg/d	1.000 (1.000, 1.000)	0.950
Thiamine, mg/d	0.820 (0.763, 0.881)	<0.001
Niacin, mg/d	0.961 (0.952, 0.970)	<0.001
Ascorbic Acid, mg/d	0.999 (0.998, 1.000)	0.002

**Table 3:** Association of riboflavin intake with suicidal ideation.

Exposure	Model 1	*p*	Model 2	*p*	Model 3	*p*
	OR (95% CI)		OR (95% CI)		OR (95% CI)	
Intake of Riboflavin	0.64 (0.59, 0.69)	<0.001	0.71 (0.65, 0.78)	<0.001	0.83 (0.77, 0.91)	<0.001
Q1	Reference		Reference		Reference	
Q2	0.62 (0.54, 0.71)	<0.001	0.77 (0.67, 0.89)	<0.001	0.96 (0.81, 1.15)	0.675
Q3	0.56 (0.44, 0.70)	<0.001	0.71 (0.56, 0.89)	0.042	1.06 (0.80, 1.42)	0.672
*p* for Trend	<0.001		0.026		0.230	
Sex						
Men	0.77 (0.68, 0.88)	<0.001	0.81 (0.71, 0.92)	0.001	0.95 (0.82, 1.11)	0.541
Women	0.59 (0.53, 0.66)	<0.001	0.65 (0.58, 0.73)	<0.001	0.78 (0.70, 0.87)	<0.001
Age						
<60	0.55 (0.48, 0.63)	<0.001	0.58 (0.50, 0.67)	<0.001	0.82 (0.69, 0.97)	0.022
≥60	0.78 (0.70, 0.87)	<0.001	0.81 (0.73, 0.90)	<0.001	0.88 (0.79, 0.97)	0.015
BMI						
<25	0.62 (0.55, 0.69)	<0.001	0.68 (0.61, 0.77)	<0.001	0.80 (0.71, 0.90)	<0.001
≥25	0.67 (0.59, 0.75)	<0.001	0.76 (0.66, 0.86)	<0.001	0.88 (0.78, 0.99)	0.049

Model 1: no covariates adjusted. Model 2: adjustments made for age and sex. Model 3: adjustments made for age, sex, marital status, educational level, household income, smoking status, drinking status, aerobic and anaerobic exercises, hypertension, dyslipidemia, diabetes mellitus, depression, body mass index, thiamine intake, niacin intake, and daily energy intake.

**Table 4:** Subgroup analysis of the association between riboflavin and suicidal ideation.

Subgroup	Suicidal Ideation [OR(95%CI)]	*p* for Interaction
Age (years)		0.2827
<60	0.97 (0.79, 1.19)	
≥60	1.14 (0.89, 1.47)	
Sex		0.0817
Men	0.88 (0.72, 1.06)	
Women	1.14 (1.00, 1.31)	
Body mass index (BMI), kg/m^2^		0.2337
<25	1.11 (0.90, 1.36)	
≥25	0.93 (0.74, 1.18)	
Aerobic Exercise		0.3587
<1 day/week	1.17 (0.85, 1.61)	
1–7 days/week	1.00 (0.83, 1.20)	
Anaerobic Exercise		0.0284
<1 day/week	1.17 (0.96, 1.44)	
1–7 days/week	0.85 (0.67, 1.08)	
Hypertension		0.6158
No	0.99 (0.79, 1.25)	
Yes	1.07 (0.87, 1.31)	
Diabetes		0.5273
No	0.98 (0.78, 1.23)	
Yes	1.07 (0.87, 1.32)	
Hyperlipidemia		0.9918
No	1.03 (0.86, 1.26)	
Yes	1.03 (0.92, 1.16)	

## Data Availability

The data analyzed in this study are openly accessible via the KNHANES website (https://knhanes.kdca.go.kr/knhanes/eng/main.do, accessed on 4 June 2024).

## References

[1] Motillon-Toudic C., Walter M., Séguin M., Carrier J.-D., Berrouiguet S., Lemey C. (2022). Social isolation and suicide risk: Literature review and perspectives. Eur. Psychiatry.

[2] Organization for Economic Cooperation and Development, Health at a Glance (2023). Organization for Economic Cooperation and Development. https://www.oecd-ilibrary.org/social-issues-migration-health/death-by-suicide-2000-and-2020-or-nearest-year_8389897a-en.

[3] Statistics Korea (2022). Suicide Trends and Responses in Korea. Statistics Research Institute. https://sri.kostat.go.kr/board.es?mid=b10104000000&bid=12046&act=view&list_no=432657&tag=&nPage=1&ref_bid=/.

[4] Franklin J.C., Ribeiro J.D., Fox K.R., Bentley K.H., Kleiman E.M., Huang X., Musacchio K.M., Jaroszewski A.C., Chang B.P., Nock M.K. (2017). Risk factors for suicidal thoughts and behaviors: A meta-analysis of 50 years of research. Psychol. Bull..

[5] Turecki G., Brent D.A. (2016). Suicide and suicidal behaviour. Lancet.

[6] Quirk S.E., Williams L.J., O’Neil A., Pasco J.A., Jacka F.N., Housden S., Berk M., Brennan S.L. (2013). The association between diet quality, dietary patterns and depression in adults: A systematic review. BMC Psychiatry.

[7] Li Y., Zhang J., McKeown R.E. (2009). Cross-sectional assessment of diet quality in individuals with a lifetime history of attempted suicide. Psychiatry Res..

[8] Nanri A., Mizoue T., Poudel-Tandukar K., Noda M., Kato M., Kurotani K., Goto A., Oba S., Inoue M., Tsugane S. (2013). Dietary patterns and suicide in Japanese adults: The Japan public health center-based prospective study. Br. J. Psychiatry.

[9] Voracek M., Tran U.S. (2007). Dietary tryptophan intake and suicide rate in industrialized nations. J. Affect. Disord..

[10] Kawachi I., Willett W.C., Colditz G.A., Stampfer M.J., Speizer F.E. (1996). A prospective study of coffee drinking and suicide in women. Arch. Intern. Med..

[11] Amerikanou C., Gioxari A., Kleftaki S.-A., Valsamidou E., Zeaki A., Kaliora A.C. (2023). Mental Health Component Scale Is Positively Associated with Riboflavin Intake in People with Central Obesity. Nutrients.

[12] McCormick D.B. (1989). Two interconnected B vitamins: Riboflavin and pyridoxine. Physiol. Rev..

[13] Al Mansoori A., Shakoor H., Ali H.I., Feehan J., Al Dhaheri A.S., Cheikh Ismail L., Bosevski M., Apostolopoulos V., Stojanovska L. (2021). The effects of bariatric surgery on vitamin B status and mental health. Nutrients.

[14] Calderón-Ospina C.A., Nava-Mesa M.O. (2020). B Vitamins in the nervous system: Current knowledge of the biochemical modes of action and synergies of thiamine, pyridoxine, and cobalamin. CNS Neurosci. Ther..

[15] Naghashpour M., Amani R., Nematpour S., Haghighizadeh M.H. (2011). Riboflavin status and its association with serum hs-CRP levels among clinical nurses with depression. J. Am. Coll. Nutr..

[16] Park S.-J., Choi J.H., Lee J.Y., Lee C., Lee H.-J. (2018). Association between nutrient intakes and prevalence of depressive disorder in Korean adults: 2014 Korean National Health and Nutrition Examination Survey. J. Nutr. Health.

[17] Kweon S., Kim Y., Jang M.-j., Kim Y., Kim K., Choi S., Chun C., Khang Y.-H., Oh K. (2014). Data resource profile: The Korea national health and nutrition examination survey (KNHANES). Int. J. Epidemiol..

[18] Lim S.-H., Kim J.-B., Cho Y.-S., Choi Y., Park H.-J., Kim S.-N. (2013). National standard food composition tables provide the infrastructure for food and nutrition research according to policy and industry. Korean J. Food Nutr..

[19] Nease D.E., Malouin J.M. (2003). Depression screening: A practical strategy. J. Fam. Pract..

[20] Hirschtritt M.E., Kroenke K. (2017). Screening for depression. JAMA.

[21] Park S.-J., Choi H.-R., Choi J.-H., Kim K.-W., Hong J.-P. (2010). Reliability and validity of the Korean version of the Patient Health Questionnaire-9 (PHQ-9). Anxiety Mood.

[22] Hawton K., van Heeringen K. (2009). Suicide. Lancet.

[23] Miyake Y., Sasaki S., Tanaka K., Yokoyama T., Ohya Y., Fukushima W., Saito K., Ohfuji S., Kiyohara C., Hirota Y. (2006). Dietary folate and vitamins B12, B6, and B2 intake and the risk of postpartum depression in Japan: The Osaka Maternal and Child Health Study. J. Affect. Disord..

[24] Rouhani P., Amoushahi M., Keshteli A.H., Saneei P., Afshar H., Esmaillzadeh A., Adibi P. (2023). Dietary riboflavin intake in relation to psychological disorders in Iranian adults: An observational study. Sci. Rep..

[25] Wu Y., Zhang L., Li S., Zhang D. (2022). Associations of dietary vitamin B1, vitamin B2, vitamin B6, and vitamin B12 with the risk of depression: A systematic review and meta-analysis. Nutr. Rev..

[26] Del Barrio A.G., de Santayana G.P., Guerrero F.R., Gonzalez P.B., Giraldo G.C., Gómez J.G., García-Unzueta M. (2022). Suicidal ideation in a sample with a first-episode of restrictive eating disorders: The role of biomarkers. J. Affect. Disord. Rep..

[27] Kim J.-M., Kang H.-J., Kim J.-W., Choi W., Lee J.-Y., Kim S.-W., Shin I.-S., Kim M.-G., Chun B.J., Stewart R. (2023). Multiple serum biomarkers for predicting suicidal behaviours in depressive patients receiving pharmacotherapy. Psychol. Med..

[28] Bremner J.D., Goldberg J., Vaccarino V. (2021). Plasma homocysteine concentrations and depression: A twin study. J. Affect. Disord. Rep..

[29] Ganji V., Kafai M.R. (2004). Frequent consumption of milk, yogurt, cold breakfast cereals, peppers, and cruciferous vegetables and intakes of dietary folate and riboflavin but not vitamins B-12 and B-6 are inversely associated with serum total homocysteine concentrations in the US population. Am. J. Clin. Nutr..

[30] Jacques P.F., Bostom A.G., Wilson P.W., Rich S., Rosenberg I.H., Selhub J. (2001). Determinants of plasma total homocysteine concentration in the Framingham Offspring cohort. Am. J. Clin. Nutr..

[31] Koweszko T., Gierus J., Zalewska A., Maciejczyk M., Waszkiewicz N., Szulc A. (2020). The relationship between suicide and oxidative stress in a group of psychiatric inpatients. J. Clin. Med..

[32] Kennedy D.O. (2016). B vitamins and the brain: Mechanisms, dose and efficacy—A review. Nutrients.

[33] Huang J., Tian L., Wu X., Yang H., Liu Y. (2010). Effects of dietary riboflavin levels on antioxidant defense of the juvenile grouper Epinephelus coioides. Fish Physiol. Biochem..

[34] Deus L.A., Neves R.V.P., Correa H.L., Reis A.L., Honorato F.S., Silva V.L., de Araujo T.B., Souza M.K., Sousa C.V., Simoes H.G. (2021). Improving the prognosis of renal patients: The effects of blood flow-restricted resistance training on redox balance and cardiac autonomic function. Exp. Physiol..

[35] Pinho R.A., Aguiar A.S., Radak Z. (2019). Effects of Resistance Exercise on Cerebral Redox Regulation and Cognition: An Interplay between Muscle and Brain. Antioxidants.

[36] Miná V., Lacerda-Pinheiro S., Maia L., Pinheiro R., Meireles C., De Souza S., Reis A., Bianco B., Rolim M. (2015). The influence of inflammatory cytokines in physiopathology of suicidal behavior. J. Affect. Disord..

[37] Serafini G., Parisi V.M., Aguglia A., Amerio A., Sampogna G., Fiorillo A., Pompili M., Amore M. (2020). A specific inflammatory profile underlying suicide risk? Systematic review of the main literature findings. Int. J. Environ. Res. Public Health.

[38] Black C., Miller B.J. (2015). Meta-analysis of cytokines and chemokines in suicidality: Distinguishing suicidal versus nonsuicidal patients. Biol. Psychiatry.

[39] Mazur-Bialy A.I., Pocheć E. (2016). HMGB1 inhibition during zymosan-induced inflammation: The potential therapeutic action of riboflavin. Arch. Immunol. Et Ther. Exp..

[40] Robinson J., Khan N., Fusco L., Malpass A., Lewis G., Dowrick C. (2017). Why are there discrepancies between depressed patients’ Global Rating of Change and scores on the Patient Health Questionnaire depression module? A qualitative study of primary care in England. BMJ Open.

[41] Choi H.S., Choi J.H., Park K.H., Joo K.J., Ga H., Ko H.J., Kim S.R. (2007). Standardization of the Korean Version of Patient Health Questionnaire-9 as a Screening Instrument for Major Depressive Disorder. J. Korean Acad. Fam. Med..

[42] Na P.J., Yaramala S.R., Kim J.A., Kim H., Goes F.S., Zandi P.P., Voort J.L.V., Sutor B., Croarkin P., Bobo W.V. (2018). The PHQ-9 Item 9 based screening for suicide risk: A validation study of the Patient Health Questionnaire (PHQ)—9 Item 9 with the Columbia Suicide Severity Rating Scale (C-SSRS). J. Affect. Disord..

[43] Esposito C.M., Ceresa A., Buoli M. (2021). The association between personality traits and dietary choices: A systematic review. Adv. Nutr..

[44] Li Y., Zhang J. (2007). Serum concentrations of antioxidant vitamins and carotenoids are low in individuals with a history of attempted suicide. Nutr. Neurosci..

